# Synthesis and Evaluation of Metal Lipoate Adhesives

**DOI:** 10.3390/polym15132921

**Published:** 2023-07-01

**Authors:** Animesh Ghosh, Konrad Kozlowski, Terry W. J. Steele

**Affiliations:** School of Materials Science and Engineering (MSE), Nanyang Technological University (NTU), Singapore 639798, Singapore; animeshg@ntu.edu.sg (A.G.); konrad001@e.ntu.edu.sg (K.K.)

**Keywords:** (±)-alpha-lipoic acid (LA), metal lipoate, electrocuring, rheology, adhesion strength

## Abstract

The development of new bioadhesives with integrated properties remains an unmet clinical need to replace staples or sutures. Current bioadhesives do not allow electronic activation, which would allow expansion into laparoscopic and robotic surgeries. To address this deficiency, voltage-activated adhesives have been developed on both carbene- and catechol-based chemical precursors. Herein, a third platform of voltage-activated adhesive is evaluated based on lipoic acid, a non-toxic dithiolane found in aerobic metabolism and capable of ring-opening polymerization. The electro-rheological and adhesive properties of lithium, sodium, and potassium salts of lipoic acid are applied for wet tissue adhesion. At ambient conditions, potassium lipoate displays higher storage modulus than lithium or sodium salt under similar conditions. Voltage stimulation significantly improves gelation kinetics to Na- and K-lipoates, while Li-lipoate is found to not require voltage stimulation for gelation. Lap shear adhesion strength on wetted collagen substrates reveals that the synthetic metal lipoates have comparable adhesion strength to fibrin sealants without viral or ethical risks.

## 1. Introduction

Bioadhesives effectively address several disadvantages of conventional suturing or stapling, such as infection risks, steep learning curves, pain, and secondary operations. The elegant design of a stimuli-activated adhesive could potentially address many unmet clinical needs within general wound closures, fixation of medical devices, and osteopathic repair [1,2,3,4,5,6]. A common design intent for this subclass of biomaterials prioritizes resorbable constituents, rapid liquid-to-solid gelation, and a mechanism to adhere to both wet and dry tissue substrates. A diverse range of bioadhesive formulations has explored two-part and light-activated cross-linking mechanisms [7,8,9,10,11]. However, the search continues for formulations that have both sufficient adhesion, resorbable metabolites, and fail-reversible adhesion. For example, biologically derived fibrin-based bioadhesives show weak adhesion strength (10^3^ to 10^4^ Pa) and could be a potential source of viral transmission (HIV, HAV, HBV, HCV, vJCD, etc.) [12,13,14,15]. Collagen and other natural-product-based bioadhesives are readily resorbed by protease degradation but require animal-derived raw materials. Collagen-based hemostats can swell with tissue compression and hence are not recommended for ophthalmic or urological surgeries [16,17]. Synthetic adhesives (cyanoacrylate, gelatin-resorcinol-formaldehyde, etc.) exhibit adhesion strength (10^4^ to 10^6^ Pa) that exceeds soft tissue cohesive strength (e.g., tissue delamination) but have the propensity to cause acute inflammation due to their reactive cross-linkers and toxic degradation products. Thus, they are contraindicated for internal tissues with most uses as a skin sealant [18,19,20,21].

An alternative design intent identifies metabolic precursors capable of reactive cross-linking under suitable stimuli exposure and degrades back to benign monomers. (±)-Alpha-lipoic acid (LA) may be one such candidate. LA has endogenous antioxidant properties and is found in some foods such as spinach, broccoli, potato, red meat, etc. [22]. Commercially prepared in bulk quantities, LA is exploited in food supplements for its anti-inflammatory and antimicrobial properties. As an essential metabolite in aerobic metabolism, LA has been applied to vitamin C and E deficiency, Alzheimer’s disease, degenerative neurons, diabetic polyneuropathy, ischemia/reperfusion injuries, and arthritis [23,24,25,26,27].

**Figure 1:**
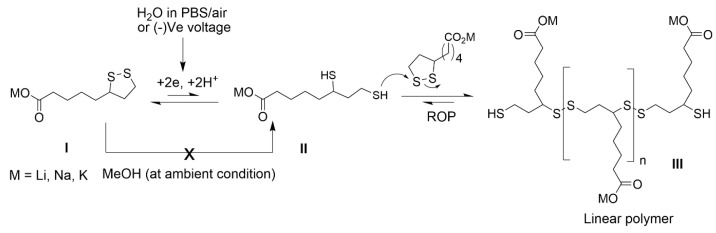
Voltage-activated polymerization of metal lipoates.

LA serves as a reactive monomer due to the strained five-membered dithiolane ring and a terminal carboxylic acid. Upon heating to 70 °C, the strained cyclic disulfide ring undergoes ring-opening polymerization (ROP), which produces polymers based on a poly-disulfide backbone [28]. An attractive feature of the poly-disulfide backbone is the ease of depolymerization through the introduction of a suitable reductant. In order to engineer LA into a suitable bioadhesive, its poor water solubility (<1 g.L^−1^) and 70 °C polymerization temperature need to be addressed [29]. Metal lipoate salts increase solubility by over 500× in aqueous media (>400 g.L^−1^) [30]. As rates of ring-opening polymerization are enhanced at higher concentrations, the following hypothesis is proposed: Anhydrous metal lipoate powders undergo rapid dissolution in neutral/basic aqueous buffers, where molar-level solutes generate dithiolane collision rates sufficient to produce poly-disulfides at ambient temperatures. If the redox reactions are dependent on electrolytes, then chemical properties that are dependent on redox kinetics (e.g., gelation kinetics) will correlate with cationic chemical properties such as ionic radius and diffusion rates. Electrochemical reduction of dithiolane yields radicals and thiols (known ROP initiators), which accelerates the liquid to solid gelation rates, as shown in Figure 1. Na-lipoate has previously been reduced to the corresponding dithiol in an aqueous solution at −0.8 V [31]. Tissue adhesion commences through protein entanglement, polysulfide interlinks, and electrostatic interactions. Herein, these hypotheses are evaluated with the synthesis of Li-, Na-, and K-lipoates. A custom electrorheology set-up evaluates the viscoelastic properties as a function of reducing cathodic voltages.

## 2. Materials and Methods

### 2.1. Materials

(±)-Alpha-lipoic acid (product no. T1395), lithium hydroxide (product no. 545856), sodium hydroxide (product no. V800383), potassium hydroxide (product no. 306568), Tris (2-carboxyethyl) phosphine (TCEP, product no. C4706), phosphate-buffered saline (PBS, product no. 806544), ethanol (product no. 459836), and pH indicator strips were purchased from Sigma-Aldrich, Singapore. Collagen film was purchased from Nippi Collagen Inc. (Tokyo, Japan). The disposable Zensor^®^ chip (product no. TE100) was purchased from Zensor R&D Company, Taipei, Taiwan.

### 2.2. General Methods

All experiments were carried out in dried glassware. Bruker AVANCE 400 MHz instruments in the deuterated solvent CD_3_OD were used to record ^1^H NMR (400 MHz) and ^13^C NMR (100 MHz) spectra. Chemical shifts are given in parts per million (ppm, d) based on the middle peak of the solvent signal (^1^H NMR, δ = 3.30; ^13^C NMR, δ = 49.15 for CD_3_OD) as an internal standard. The J values are given in Hz. Multiplicities are indicated as ‘s’ (singlet), ‘d’ (doublet), ‘t’ (triplet), ‘q’ (quartet), ‘m’ (multiplet), and ‘brd’ (broad doublet). Melting point measurements were performed using a PerkinElmer DSC 8500. All reactions were carried out under nitrogen. The evaporation of solvents was performed at reduced pressure using a rotary evaporator. All reagents and solvents were purchased from Aldrich and used without further purification unless otherwise indicated.

### 2.3. Synthesis of Metal Lipoate Salts

A solution of LiOH (116 mg, 4.85 mmol) in EtOH (15 mL) was added dropwise into a solution of lipoic acid (1.0 g, 4.85 mmol, 1.0 equiv.) in EtOH/H_2_O (10 mL/2 mL) taken in a Schlenk flask under N_2_ atmosphere at room temperature. Nitrogen was bubbled through the mixture for 5 min before it was heated at 80 °C for 1 h. The reaction mixture was cooled to room temperature, and ethanol was evaporated under reduced pressure to obtain minimum volume. Acetone (40 mL) was added and stirred vigorously for 1 h. The precipitate was filtered and dried (3 h) in a high vacuum to afford compound I (463 mg, 90%) as a light-yellow solid. Mp: 223 °C., ^1^H NMR (400 MHz, CD_3_OD) δ 3.62–3.51 (m, 1H), 3.21–3.01 (m, 2H), 2.51–2.38 (m, 1H), 2.20–2.10 (m, 2H), 1.93–1.82 (m, 1H), 1.76–1.57 (m, 4H), 1.52–1.39 (m, 2H); ^13^C NMR (100 MHz, CD_3_OD) δ 182.82, 57.86, 41.43, 39.45, 39.12, 36.09, 30.57, 27.60.

Na-lipoate was prepared following the same procedure as described for the synthesis of Li-lipoate. Mp: 257 °C., ^1^H NMR (400 MHz, CD_3_OD) δ 3.63–3.50 (m, 1H), 3.21–3.01 (m, 2H), 2.51–2.38 (m, 1H), 2.17–2.10 (m, 2H), 1.94–1.81 (m, 1H), 1.77–1.56 (m, 4H), 1.52–1.38 (m, 2H); ^13^C NMR (100 MHz, CD_3_OD) δ 182.90, 57.87, 41.43, 39.44, 39.21, 36.10, 30.59, 27.66.

K-lipoate was prepared following the same procedure as described for the synthesis of Li-lipoate. Mp: 244 °C., ^1^H NMR (400 MHz, CD_3_OD) δ 3.63–3.49 (m, 1H), 3.23–2.99 (m, 2H), 2.52–2.36 (m, 1H), 2.21–2.09 (m, 2H), 1.94–1.81 (m, 1H), 1.77–1.56 (m, 4H), 1.53–1.37 (m, 2H); ^13^C NMR (100 MHz, CD_3_OD) δ 182.84, 57.87, 41.43, 39.44, 39.21, 36.10, 30.59, 27.66.

### 2.4. Size-Exclusion Chromatography (SEC)

Size-exclusion chromatography (SEC) was used to measure the masses of metal lipoate polymers (Shimadzu SIL 20AC, Singapore). The instrument has three detectors: UV/Vis, multi-angle light scattering (MALS, Wyatt Technology Corporation, Santa Barbara, CA, USA), and refractive index (RID, 10A). A PLGel 5 mm 1000Å column in a 40 °C oven was utilized in SEC along with 100% tetrahydrofuran (THF) eluent at a flow rate of 1 mL.min^−1^. The polymeric metal lipoates showed poor solubility in THF. Therefore, polymeric metal lipoates were first dissolved in MeOH, to which an equal volume of THF was added, making MeOH-THF (1:1) at a concentration of 0.5 mg/mL. It was observed that the addition of THF turned the methanolic solution a little cloudy, probably due to the formation of colloidal particles typical of polyelectrolyte materials in aprotic organic media, which was filtered with a 0.2 μm syringe and injected (50 μL). Dn/dc of 0.107 was used for all the metal lipoates, calculated using the RI of lipoic acid as 1.524. The average molecular weights (M_n_ and M_w_) of metal lipoates are reported in Table S1.

### 2.5. Cyclic Voltammetry (CV)

Cyclic voltammetric measurements were performed using a Metrohm Autolab PGSTAT302N potentiostat in a three-electrode set-up. A 3 mm diameter planar glassy carbon disk (Metrohm) was used as a working electrode in conjunction with a platinum plate counter electrode (Metrohm) and a Ag/AgCl reference electrode (filled with 3.0 M KCl solution). The cyclic voltammetric data were recorded in PBS at a scan rate of 0.1 V/s. All voltammetric experiments were conducted under an argon atmosphere, at room temperature, in a Faraday cage. Prior to each scan, the working electrode was cleaned by polishing with an alumina oxide (grain size 0.3 mm) slurry on a Buehler Ultra-pad polishing cloth, rinsed with ethanol, and then dried with a lint-free tissue.

### 2.6. Preparation of Metal Lipoate Formulation

Metal lipoates display higher solubility in aqueous media than in organic media such as methyl alcohol, ethyl alcohol, or dimethyl sulfoxide. All the formulations were prepared in PBS (pH~7.2). A measured volume of PBS was added to the powdered Li-lipoate (85 mg Li-lipoate/100 mL PBS for 4M solution) in a vial and vortexed for 3–4 min to obtain a clear solution (2M–5M concentration). Na- and K-lipoate formulations were prepared following the same procedure. The 4M formulation of Li-/Na- and K-lipoate prepared in PBS displayed pH~9–10 on pH indicator strips.

### 2.7. Electrorheology Studies of Li-, Na, and K-Lipoate

The Anton Paar MC102 rheometer equipped with a portable potentiostat power supply (pocketSTAT, Ivium Technologies, Eindhoven, The Netherlands) was utilized to record the viscoelastic properties of the metal lipoate adhesive. A disposable polypropylene-based Zensor^®^ chip, embedded with a 3-mm diameter glassy carbon (GC) as a working electrode (WE), an outer annular crescent GC as a counter electrode (CE), and a Ag/AgCl pellet as a reference electrode (RE), served as the rheometer baseplate. Oscillatory tests were performed at the measuring gap of 0.3 mm, an amplitude of 10%, and a frequency of 1 Hz with a rheometer ceramic probe parallel plate 10 mm in diameter (PP10 ceramic, Anton PAAR, USA). Li-, Na-, or K-lipoate was dissolved in PBS (4M concentration) and mixed well to obtain a clear solution. The potentiostat applied different voltages (−3.0 V, −5.0 V) to the lipoate adhesive formulation for 28 min. All samples were evaluated in triplicate, and minimum torque measurements were 20 Nm. Voltage was applied after two minutes of steady state shear. A dynamic mechanical analysis calculated loss modulus (G″) and storage modulus (G′) with a data sampling frequency of 1 Hz. A steady state rotational viscosity test was performed at a fixed shear rate of 62.8 rad.s^−1^ (10 s^−1^ @ ¾R). Amplitude sweeps determined the shear strain at break, yield stress, linear viscoelastic range, or a combination thereof of the cured Li-/Na-/K-lipoate adhesive.

### 2.8. Lap Shear Adhesion on Collagen Substrates

Lap shear adhesion studies were performed according to the ASTM standard F2255-05. Shear adhesive strength failure was measured using a tensile tester (Chatillon Force Measurement Products, Singapore) using a 50 N loading cell with a constant linear speed of 3 mm.min^−1^. Before applying the metal adhesive, the collagen strip is dipped for 5 min into PBS, removed, and the superficial water is absorbed with tissue paper [32,33]. Lipoate adhesive (50 mg, 4M formulation) is sandwiched between the two wet collagen films (length—20 mm, width—20 mm, thickness—0.01 mm) and secured with paper clips (~1 N compressive force). After 30 min of curing at room temperature, a lap shear adhesion test was performed. Adhesive shear strength at failure was determined by the maximum load (newtons) divided by the surface area (square meters).

### 2.9. Peel Adhesion on Ex Vivo Skin Substrates

The pig skin was dipped sequentially into deionized water (for 1 h), followed by PBS for another 1 h. The surface water was wiped with tissue paper. The 4M formulation of Li-lipoate adhesive was applied onto a pig skin (length—30 mm, width—20 mm) and sandwiched with a hydrated collagen film, thus forming a pig skin/Li-lipoate adhesive/collagen sandwich structure, which was placed in between two glass slides and fastened with paper clips. The adhesive was allowed to cure for 30 min at ambient temperature (24 °C) or at 37 °C, and then the peel adhesion strength was measured on a tensile tester (Chatillon Force Measurement Products, USA) pulling the sample at the speed of 10 mm min ^−1^ using a 50 N load cell. Peel adhesion strength was determined following previously established guidelines (Peel strength = N/width) [34].

### 2.10. Statistical Analysis

All data are presented as mean values ± standard deviation (*n* = 3). One-way ANOVA statistical analysis was performed by Tukey’s comparison, and *p* < 0.05 was set as significant in all the tests. All of the calculations and graphs were produced using OriginPro 2020 64-bit software.

## 3. Results

### 3.1. Scope of Metal Lipoate Adhesives

Three metal salts of alpha-lipoic acid (Li, Na, and K) were synthesized to increase aqueous solubility from 3 mM up to 5 M. NMR studies (^1^H/^13^C NMR) confirm that metal lipoate salts do not have any polymeric impurity (Figure S2). An attempt was made to investigate the average molecular weight of metal lipoate polymers with size-exclusion chromatography (SEC) using THF as the mobile phase. The molar mass of all the metal lipoate polymers was found to be greater than 100 kDa, which was speculated to be due to the aggregation in organic media (Table S1). The aqueous mobile phase could not be used due to the depolymerization of metal lipoate polymers upon aqueous dilution. The synthesized metal lipoates are yellow solids and have >6 months of shelf stability when refrigerated. Liquid properties (apparent viscosity) were first screened to determine the highest concentration at which the formulation remains viscous and transparent. Li-lipoate was used as the model compound with a solubility limit of up to 4M concentration. Further increasing the concentration (5M) turned the formulation turbid. Cyclic voltammetry evaluated the reduction potential of the metal lipoate disulfide bonds. The high ionic concentrations were exploited to carry electric currents, with the prediction that reducing voltages would shift the precursor equilibrium to the reactive dithiol monomer (ROP initiator) and accelerate the polymerization, as shown in Figure 1. If the latter is true, electrorheology measurements should reveal shorter gelation times (tan δ ≤ 1), kinetic acceleration of the complex (G*), and shear storage modulus (G′) or a combination thereof as compared to 0 V control. A custom electro-rheometer integrated a disposable 3-electrode chip as the rheometer baseplate. Thus, real-time measurements of complex, storage, and loss moduli were recorded with respect to voltage, cation, and time. If gelation was observed, subsequent amplitude sweeps determined the shear stress, modulus, yield strain, and shear adhesive strength of the lipoate formulation cured for 30 min. If there was no gelation, frequency sweeps evaluated for gelation under increasing shear rates. The ^1^H NMR analysis of the adhesive matrix after 10 min of curing measured the voltage-activated ROP kinetics as compared to ambient conditions (Figure S8). Lipoate monomers were stable in an alcoholic solution, arresting the polymerization process after 10 min of curing. Preliminary demonstrations of wet tissue adhesion were applied to the reinforced collagen films (Nippi sausage casings) to evaluate lap shear adhesion strength. Peel adhesion strength on pig skin evaluated the suitability of metal lipoates for application as removable skin tapes. Addition of the reducer TCEP evaluated on-demand depolymerization.

### 3.2. Optimal Metal Lipoate Dissolution at 4 mol/L

**Figure 2:**
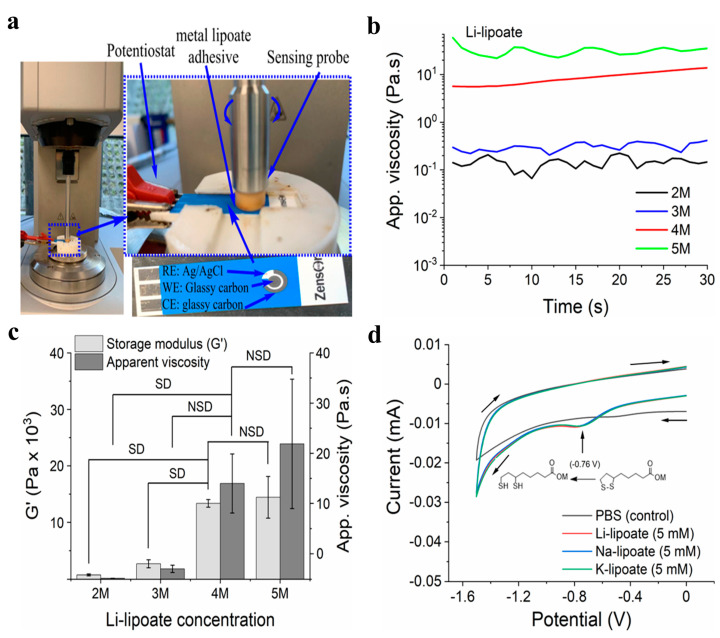
(**a**) Real-time electrorheology of Li- and Na-lipoate adhesive (**b**) Representative apparent viscosity graphs of 2M–5M Li-lipoate formulations at a fixed shear rate of 10 s^−1^ / 62.8 rad.s^−1^. (**c**) Storage modulus and apparent viscosity of Li-lipoate at different concentrations (2M–5M) without voltage application; data presented as mean ± standard deviation, *n* = 3, *p*-values are calculated using one-way ANOVA with Tukey test, SD (statistical difference) = *p <* 0.05. (**d**) Cyclic voltammogram of metal lipoates in PBS (5 mM) using 3 mm diameter GC working electrode vs. Ag/AgCl at 298 ± 2 K in a Faraday cage. Scan rate −0.1 V.s^−1^.

Li-lipoate powder readily dissolved in PBS in less than 5 min to obtain 2M–5M Li-lipoate formulations. This allowed for the rapid preparation of 1–2 mL aliquots to use as needed. Figure 2b exhibits that the apparent viscosity of 2M–4M concentrations remained under 10 Pa.s, but a critical point was exceeded for 5M with a viscosity of 35 Pa.s—similar to the flow of ketchup. The screening of lipoate formulations revealed that the 4M solution had the highest G′ with the lowest concentration, as shown in Figure 2c. The 4M solution was chosen for subsequent rheological and lap shear/peel adhesion strength investigations. Overall, all solutions were viscous after dissolution but permitted application with 1–5 mL disposable syringes with large bore needles (>16 G).

### 3.3. Na- and K-Lipoates Remained Viscous but Li-Lipoate Gelled within 5 Min

Any liquid glue requires an estimation of the operation window for viscous placement and manipulation. Evaluation of the viscoelastic profile through rheometry allowed tandem experiments under constant shear (apparent viscosity, 10 s^−1^ @ 3/4R), followed by oscillatory strains. Immediately after dissolution, a 4M solution was placed between an upper moving plate (rheometer probe) and a lower stationary plate (3-electrode chip). Rotational shear fissured most intermolecular attractive forces (e.g., H-bonds). Li-, Na-, and K-lipoate formulations, upon reaching equilibrium, displayed an apparent viscosity of (12 ± 3) Pa.s, (0.50 ± 0.20) Pa.s, and (0.45 ± 0.15) Pa.s, respectively (Figure S4a–c). Complex viscosity profiles of all formulations displayed an evolving dynamic viscosity, indicating the onset of disulfide polymerization (Figure S5a–d). Li-lipoate showed signs of gelation in under 5 min, while Na- and K-lipoate formulations remained viscous at this time. Gelation time was defined as the viscous (G″ > G′) to solid crossover time point where G″ = G′.

### 3.4. Electric Field Accelerated Gelation Time and Increased G′ of K- and Na-Lipoates

**Figure 3:**
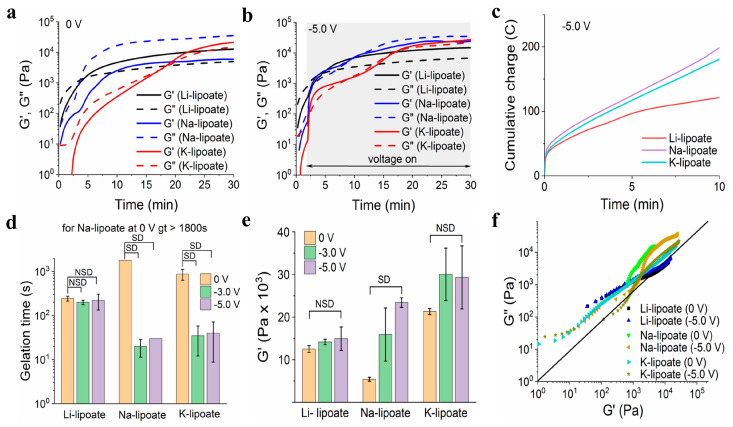
Variation of storage and loss modulus (G′ and G″) of metal lipoate adhesive 4M formulation at (**a**) 0 V and (**b**) −5.0 V over 30 min. (**c**) Representative cumulative charge vs. time graph of the metal lipoates for the first 10 min curing at −5.0 V. (**d**) Gelation time (gt) of 4M formulation of metal lipoates at different voltages (0 V, −3.0 V, and −5.0 V); data presented as mean ± standard deviation, *n* = 3, *p*-values are calculated using one-way ANOVA with Tukey test, NSD (no statistical difference) = *p <* 0.05. (**e**) Storage modulus (G′) of 4M formulation of metal lipoates at different voltages (0 V, −3.0 V, and −5.0 V); data presented as mean ± standard deviation, *n* = 3, *p*-values were calculated using a one-way ANOVA with Tukey test, NSD (no statistical difference) = *p <* 0.05. (**f**) Vector graph of metal lipoates at different voltages (0 V, −5.0 V).

Gelation time and complex modulus were further evaluated under the influence of a cathodic electric field (Figure 2a and Figure S13) [35]. Sheared lipoates were subjected to an electric field of −3.0 V and −5.0 V and compared to 0 V (control). The negative magnitude indicated cathodic reduction at the working electrode (largest). If the redox hypothesis described above applies, the gelation time should be significantly shorter and correlate with the voltage value. No gas evolution was observed during cathode activation, suggesting other lower voltage processes were not occurring. Water electrolysis occurred at −1.2 V with gas evolution. Under ambient conditions, all three metal lipoate formulations underwent polymerization, attributed to the disulfide ring opening, as shown in Figure 1. However, for Li-lipoate (4M), gelation occurred within 4.1 ± 0.5 min and for K-lipoate at 14.6 min (±4.0 min). Na-lipoate did not display enough polymerization to reach gelation within 30 min, but storage and loss modulus continued to escalate (Figure 3a). Cross-linking kinetics of Na- and K-lipoates were accelerated by the application of voltage, with gelation times of 30 s (±0 s at −5.0 V) and 40 s (±31 s at −5.0 V), respectively, after cathodic activation (Figure 3b,d). Voltage-stimulated curing of the Li-lipoate formulation showed no increase of storage modulus (0 V vs. −5.0 V has NSD, *p* < 0.05) (Figure 3e), even though ^1^H NMR displayed an acceleration of monomer consumption under voltage; 5.5% @ 0 V vs. 12.7% @ −5.0 V after 10 min (Figure S8). Na-lipoate showed a significant increase in storage modulus (0 V vs. −5.0 V, *p* < 0.05), which is summarized in Figure 3e. Complex modulus (G*) comparisons with and without voltage displayed a clearer comparison of how voltage accelerates curing with little to no improvement in modulus after 25 min (Figure S5a–d).

### 3.5. Cured Li-Lipoate Displayed the Highest Resilience under Shear Load

**Figure 4:**
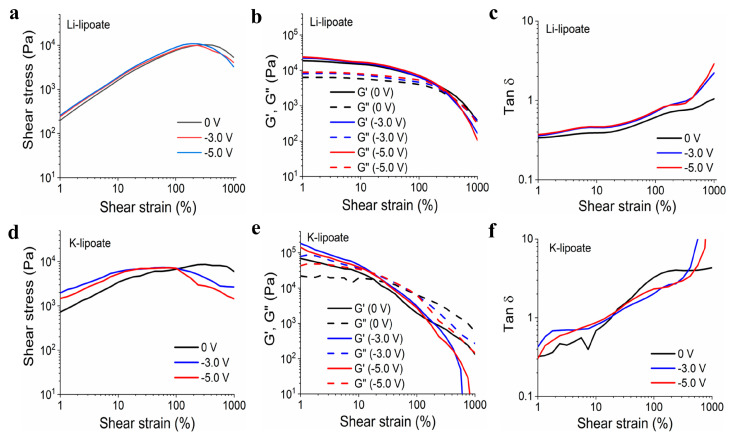
(**a**) Li-lipoate shear strain vs. shear stress at different voltages (0 V, −3.0 V, and −5.0 V). (**b**) Li-lipoate shear strain vs. modulus (G′ and G″) at different voltages (0 V, −3.0 V, and −5.0 V). (**c**) Li-lipoate shear strain vs. Tan d (G′ and G″) at different voltages (0 V, −3.0V, and −5.0 V). (**d**) K-lipoate shear strain vs. shear stress at different voltages (0 V, −3.0 V, and −5.0 V). (**e**) K-lipoate shear strain vs. modulus at different voltages (0 V, −3.0 V, and −5.0 V). (**f**) K-lipoate shear strain vs. Tan d (G′ and G″) at different voltages (0 V, −3.0 V, and −5.0 V).

Due to their spontaneous gelation properties, Li- and K-lipoate were evaluated by amplitude sweep under a strain range of 1 to 1000%. Yield stress indicated the load-bearing capacity before solid-to-viscous (plastic) deformation and generally correlated with shear adhesion strength. The yield stresses exhibited by Li-lipoate are 9.9 kPa (±0.4 kPa at 0 V), 10.4 kPa (±0.4 kPa at −3.0 V), and 11.1 kPa (±2.6 kPa at −5.0 V), respectively (Figure 4a). K-lipoate formulations displayed lower values of yield stress: 7.4 kPa (±1.4 kPa at 0 V), 6.9 kPa (±1.8 kPa at −3.0 V), and 8.9 kPa (±2.4 kPa at −5.0 V) (Figure 4d). The yield stress of Li-lipoate adhesive, though it displayed a positive correlation with the applied voltage (Pearson’s *r* = 0.98), did not have a statistical difference (Figure S6a). Yield stress values of K-lipoate adhesive without or with voltage did not display any correlation (Pearson’s *r* = 0.51) and no statistical difference (Figure S6a). This suggests cathodic activation does not influence the degree of polymerization but only the time to reach equilibrium. Na-lipoate adhesives exhibited viscous behavior at the end of 30 min curing. As gelation was not observed, frequency sweep evaluated the presence of viscous to elastic transitions under increasing shear rates. Figure S6b displays no shear-induced gelation up to 100 Hz.

### 3.6. All Three Metal Lipoates Showed Comparable Adhesion Strength on Wet Tissue Mimics

**Figure 5:**
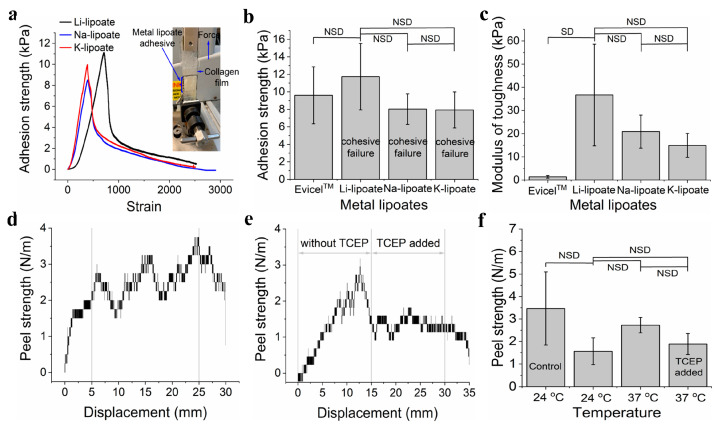
(**a**) A representative graph for the lap shear adhesion strength test of three metal lipoates (4M formulation) after 30 min curing at room temperature (0 V). (**b**) Comparison of lap shear adhesion strength of the three metal lipoates (4M formulation) after 30 min curing at room temperature (0 V); data presented as mean ± standard deviation, *n* = 3, *p*-values were calculated using a one-way ANOVA with Tukey test, NSD (no statistical difference) = *p <* 0.05. (**c**) Comparison of lap shear modulus of the toughness of the three metal lipoates (4M formulation) after 30 min curing at room temperature (0 V); data presented as mean ± standard deviation, *n* = 3, *p*-values were calculated using a one-way ANOVA with Tukey test, NSD (no statistical difference) = *p <* 0.05. (**d**) A representative graph for the peel adhesion strength of Li- lipoates (4M formulation) after 30 min curing at 37 °C temperature (0 V). (**e**) A representative graph for the peel adhesion strength test of Li- lipoates (4M formulation, 30 min curing at 37 °C) before and after adding TCEP solution (50 mM aqueous solution; 0 V). (**f**) Comparison of peel adhesion strength of Li-lipoates (4M formulation) for curing at 24 °C and 37 °C before and after adding TCEP solution (50 mM aqueous solution) (control = cellophane tape); data presented as mean ± standard deviation, *n* = 3, *p*-values were calculated using a one-way ANOVA with Tukey test, NSD (no statistical difference) = *p <* 0.05.

Lap shear adhesion measures the load capacity between adhesive and substrate. Adhesion strength indicates the shear stress at the failure point (maximum load by the surface area, N.m^−2^). The experimental set-up for the lap shear adhesion is shown in Figure 5a. Metal lipoate adhesive (4M concentration) was applied on the 2 cm × 2 cm area between two wet collagen films. Curing conditions were room temperature for 30 min before load evaluation. Representative load-displacement curves of metal lipoates are displayed in Figure 5a. Three metal lipoates displayed comparable modulus of toughness (Figure 5c). A comparison of the adhesion strength of the three metal lipoates exhibited that the adhesion strength for Li-, Na-, and K-lipoates were (9.7 ± 1.4) kPa, (8.4 ± 1.0) kPa, and (7.9 ± 2.0) kPa, respectively (Figure 5b).

### 3.7. Peel Adhesion Strength Can Be Reduced by Adding TCEP Solution

Peel adhesion strength was performed by sandwiching the Li-lipoate adhesive between pig skin and a hydrated collagen film. Figure 5d shows the representative peel adhesion test graphs for curing the adhesive at 37 °C for 30 min. Peel adhesion strength at ambient temperature was 1.56 N.m^−1^ (±0.59 N.m^−1^) and at 37 °C was 2.72 N.m^−1^ (±0.34 N.m^−1^). Thus, there was no significant increase in peel adhesion strength by curing the adhesive at 37 °C. Peel strength is similar to commercial pressure-sensitive cellophane tapes of 3.4 N ± 1.6 N.m^−1^. All specimens displayed a cohesive failure. The addition of TCEP evaluated reversible adhesion by reducing and cleaving disulfide bonds into thiols (Figure 5e,f). Peel adhesion strength dropped across some samples but was not consistent across all samples; thus, no statistical difference was found. From Figure 5f, it is seen that peel adhesion strength dropped after the addition of the TCEP solution.

## 4. Discussion

(±)-Alpha-lipoic acid’s poor water solubility (0.62 g.L^−1^ as the free acid) and heat-activated polymerization have prevented its adhesive application. To overcome this limitation, LA has previously been grafted on redox-active macromolecules or particles yielding bioadhesive hydrogels. Chai et al. prepared LA-silver nanoparticle (AgNP) [36] and polydopamine (PDA)-poly (thioctic acid) (PTA) hydrogels [37] that displayed 10–50 kPa adhesion strength on porcine skin but required extended curing times of 6 h or more. Shao et al. prepared a thioctic acid-tannic acid-phytic acid hydrogel that also adhered to porcine skin but required an even longer 24 h incubation period [38]. Films of sodium lipoate have been formed by driving ring-opening polymerization through water evaporation [30]. This elegant method drives polymerization to completion and yields stiff plastic films (>100 MPa Young′s modulus) but requires 2–6 h of preparation. Zhang et al.’s investigation also displayed a high sensitivity to humidity, which is a probable cause of variability among batch preparations and viscoelastic properties. Herein, the viscoelastic behavior of three metal lipoates in aqueous media is evaluated after aqueous dissolution and voltage activation.

(±)-Alpha-lipoic acids (LA) were prepared into cationic lipoate salts in a one-step synthetic procedure that expands the usage for lipoates, especially for applications requiring rapid dissolution. Other potential benefits include (i) a biocompatibility profile similar to LA, (ii) voltage-tunable material properties, and (iii) depolymerization through disulfide cleavage strategies. No polymerization was observed on ^1^H/^13^C NMR spectra of metal lipoates, despite heating at 80 °C during synthesis, though LA reportedly polymerizes at 70 °C [28]. This is because the carboxylic acid functional group (CO_2_H) in the presence of alkali metal hydroxides (LiOH/NaOH/KOH) converts to the corresponding salts (CO_2_M; M = Li, Na, K), providing stability to metal lipoates in alcoholic solutions at high temperature. In an alcoholic solution, metal lipoates remain monomers for months without decomposition or polymerization (Figure S7). It is observed that the rate of ROP increases as the concentration of metal lipoates increases (Figure 2c). The solubility of metal lipoates in MeOH is much lower than that in the PBS buffer (Figure S1). Thus, it is speculated that a low concentration of metal lipoates in an alcoholic solution retards the formation of the linear polymer via the ROP mechanism. The solubility limit of each metal lipoate in PBS was investigated under limiting conditions (Figure S1).

The cyclic lipoate undergoes ROP to form a linear polymer, though the reaction is equilibrium dependent and does not favor polymerization [30]. Our hypotheses predicted that a reductive voltage would accelerate the initiation and shift equilibrium towards polymerization (Figure 1). The strategy relies on cathodic voltage to accelerate ROP kinetics by increasing the concentration of dithiol intermediate (initiator). Once the dithiol intermediate is formed, it initiates ROP by reacting with local metal lipoate monomers. Inexplicably, the hypotheses were cation dependent. An optimization study with Li-lipoate adhesive at ambient conditions revealed syringable liquid behavior and the highest storage modulus at 4M concentration (Figure 2b,c). In order to compare the rheological and mechanical properties, all studies of Na- and K-lipoates were carried out with the same molar concentration (4M). Electrorheology revealed that cathodic stimulation accelerated the gelation and viscoelastic properties of sodium and potassium lipoate salts. Li lipoates have inherent high polymerization kinetics, such that there was no improvement with applied cathodic electric fields after 25 min. Generally, the electric fields had the greatest effect in the following order sodium > potassium > lithium. In contrast to lithium, sodium lipoates required an electric field to reach gelation; otherwise, they remained viscous. A comparison of storage modulus also suggests a higher polymerization equilibrium under applied voltage. Na- and K-lipoates displayed a significant increase in G′ after 25 min of voltage exposure. Thus, the prepared metal lipoates allow diverse methods for tuning viscoelastic properties: (1) through the passive dissolution of lithium lipoates, (2) voltage activation of sodium/potassium, and (3) selecting a 2M–5M concentration range. For the first time, LA salts demonstrate utility for rapid gelation biomaterials, such as non-toxic bioadhesives. Qualitative visual observations found K-lipoate powder to be more hygroscopic than Li-lipoate, indicating another benefit of Li salt. The combination of concentrated cationic lipoates and cathodic electric fields achieves a higher consumption of monomer but is still far from unity. A ^1^H NMR analysis after 30 min of 4M dissolution, voltage activation, or both revealed that only 34–38% of monomer converts to linear polymeric structures (Figures S9–S11). All cationic lipoates remained monomers in anhydrous alcoholic solutions for up to 6 months of storage at RT (see Supplementary Information). An aqueous/alcohol mixture could be a potential delivery vehicle, providing molar solutes and a rapid method of evaporation/film formation, albeit with considerable VOC formation. The Li-lipoates displayed unique viscoelastic character, where comparison of rheology displayed rapid gelation within minutes regardless of voltage stimulation. The exact nature of this phenomenon remains unknown, but we list the following suppositions: (1) Lithium ions induce/accelerate redox cleavage or alkaline hydrolysis of the disulfide bond, although the latter takes hours at room temperature [39]. Either process produces the required initiator. (2) Lithium ions and complexes are known to catalyze ring-opening reactions, mostly for ester and silane polymers [40]. (3) Investigation of Li-S batteries has found species of Li_2_S_2_ and L_2_S_4_, speculating potential redox intermediates for disulfide cleavage [41]. Either way, the addition of Li cation produces shear moduli comparable to the voltage-activated Na/K cations.

Lap shear adhesion was evaluated with wet collagen films (e.g., synthetic sausage skins), which is a convenient animal-free mimic of wet tissue substrates. The primary adhesion mechanism is mechanical interlocking and polar/H-bonding with the amino acid network. The collagen has little to no cysteine content. Thus, covalent interactions are unlikely [32]. However, in tissues where cysteine is present, disulfide linkage could occur. The cohesive strength is due to the polymer entanglement and electrostatic interaction between water molecules and carboxylate groups (Figure S12). The average lap shear adhesion strength of metal lipoates was relatively weak at ~8–12 kPa (Figure 5b), which is comparable to fibrin glue hydrogels (e.g., Evicel^TM^). This limits the application of metal lipoate adhesives for topical use at this stage of development. Furthermore, aqueous depolymerization further restricts their internal application under wet conditions (surgical procedure). Adhesion and material properties at this stage require further improvement before dedicated studies on in vitro and in vivo testing. Further design improvement, such as incorporating interpenetrating networks, will enhance both rheological and mechanical properties and is the focus of our future work. It was found that there was no significant change in peel adhesion strength by increasing temperature from 24 °C to 37 °C, indicating not much change in the degree of polymerization.

Some limitations on the cationic lipoates are noted. Viscoelastic properties of metal lipoates varied from batch to batch, despite a reproducible ^1^H/^13^C chemical characterization. For example, some Li-lipoate powders had considerably higher shear moduli of 190 kPa upon dissolution (data not shown), but these stiffer hydrogels could not be reliably reproduced. The data reported herein was the most reproducible between three separate synthetic batches and after careful attention to anhydrous storage conditions and dissolution practices. The ratio of free monomers remains at molar-level hypertonicity, which will cause local dehydration if applied to wet tissue surfaces. The normal levels of Li^+^, Na^+^, and K^+^ in blood are <0.005 mmol/L, 136–145 mmol/L, and 3.6–5.2 mmol/L, respectively. Assuming a ≤ 1g topical application of 4M metal lipoate adhesive, this would increase cation levels by a maximum of 4 mmol under an unlikely instant dissolution scenario. This may increase metal ion concentration of 0.8 mmol/L in the blood (5 L blood/person). Additionally, lithium drugs are used as antimanic medication, and therapeutic toxicity is found at the level of >1.5 mmol/L [42,43]. Potassium is found in many foods and is essential for the heartbeat (K^+^ channel), and thus KCl medication is given to treat the low level of potassium in the blood. In the case of Na^+^ and K^+^, an increase of 0.8 mmol/L concentration is within the normal physiological fluctuations. The voltage and current (−5 V DC at less than 1 mA) used in this study are considered safe if topically applied [44]. This avoids the electrostimulation ranges (>5 mA levels of 10–1000 Hz alternating currents) that induce muscle contraction or neuron stimulation [45,46].

Our future work will address methods to further shift metal lipoate equilibrium beyond 35% monomer consumption, tune shear moduli between 10–100 kPa (range of most soft tissues), and attain lap shear adhesion in excess of 20 kPa (similar to commercial skin tapes).

## 5. Conclusions

This paper describes the synthesis of three alkali metal salts of LA (lipoate) and the evaluation of their rheological and mechanical properties at ambient or under cathodic activation in PBS. Metal lipoates have an improved aqueous solubility (up to 5M) and can be prepared in 5 min. Alkali metal salts display shelf stability of > 6 months in powdered or in alcoholic solutions. At ambient conditions, the optimized formulation (4M solution) of Li-lipoate gels is within 5 min, while Na- and K-lipoate remain viscous with an escalating complex modulus. The application of cathodic voltage significantly accelerates the gelation time of Na- and K-lipoate adhesives, though it remains unchanged for Li-lipoate adhesive. On a wet collagen film substrate, all three metal lipoates display comparable lap shear adhesion strength (~8–12 kPa), similar to that of fibrin glue hydrogels. Peel adhesion strength, performed on pig skin and a wet collagen film, is not significantly different at 37 °C as compared to ambient conditions. The findings provide a platform for spontaneous or voltage-activated gelation of metal lipoate-based hydrogels. It is anticipated that the metal lipoates presented here will find application in ionic hydrogels, interpenetrating networks, or bioadhesive engineering.

## Data Availability

All data included in this study are available upon request to the corresponding author.

## Supplementary Materials

The following supporting information can be downloaded at: https://www.mdpi.com/article/10.3390/polym15132921/s1, Figure S1: Synthesis of Li-, Na, and K-lipoate; Figure S2: ^1^H and ^13^C NMR spectra of metal lipoates; Figure S3: Melting point of metal lipoates; Figure S4: Apparent viscosity of metal lipoate formulations (4M solution); Figure S5: Complex modulus (G*) of metal lipoates; Figure S6: A comparison of yield stress of Li- and K-lipoates at different voltages; frequency Vs shear stress, frequency Vs modulus, and frequency Vs Tan d of Na-lipoate at different voltages (0V, −3.0 V, and −5.0 V); Figure S7: ^1^H NMR of metal lipoates in alcoholic solution up to 6 months; Figure S8: ^1^H NMR of Li-lipoate after 10 min curing at 0 V and −5.0 V; Figure S9: ^1^H NMR spectra of Li-lipoate before and after curing (30 min) at ambient condition; Figure S10: ^1^H NMR spectra of Na-lipoate before and after curing (30 min) at ambient condition; Figure S11: ^1^H NMR spectra of K-lipoate before and after curing (30 min) at ambient condition; Figure S12: A schematic diagram of metal lipoate tissue adhesion; Figure S13: A schematic diagram of Zensor electrode set-up in rheometer; Table S1: Results of average molecular weight determination by SEC.
