# Supplementary material for: Synthesis and Evaluation of Metal Lipoate Adhesives
Source: Polymers (Basel). 2023 Jul 1;15(13):2921. doi: 10.3390/polym15132921 (PMC10347271; doi:10.3390/polym15132921)
Supplement: Supplementary file 1 [file polymers-15-02921-s001.zip › polymers-2413458-supplementary.pdf]

Supporting Information

Synthesis and Evaluation of Metal Lipoate Adhesives

Animesh Ghosh, Konrad Kozlowski, Terry W.J. Steele*

School of Materials Science and Engineering (MSE), Nanyang Technological University (NTU), Singapore 639798, Singapore; animeshg@ntu.edu.sg (A.G.); konrad001@e.ntu.edu.sg (K.K.)

*Corresponding to: Terry W. J. Steele (e-mail: wjsteele@ntu.edu.sg)

Table of Contents

Results of average molecular weight determination by SEC (Table S1) ………………..… 2

Synthesis of Li-, Na, and K-lipoate (Fig. S1) ……………………………………...…. 2

^{1}H and ^{13}C NMR spectra of metal lipoates (Fig. S2) ………………………..….....…. 4

Melting point of metal lipoates (Fig. S3) ……………………………………….…...… 6

Apparent viscosity of metal lipoate formulations (4M solution) (Fig. S4) …………...…. 6

Complex modulus (G*) of metal lipoates (Fig. S5) ……….……………………….…… 7

A comparison of yield stress of Li- and K-lipoates at different voltages (Fig. S6a) …..…. 8

Frequency Vs shear stress, frequency Vs modulus, and frequency Vs Tan δ of Na-lipoate at different voltages (0V, −3.0 V, and −5.0 V). (Fig. S6b-d) …………………..……....…. 8

^{1}H NMR of metal lipoates in alcoholic solution up to 6 months (Fig. S7) ……….….…. 10

^{1}H NMR of Li-lipoate after 10 min curing at 0 V and −5.0 V (Fig. S8) ……….….…. 11

^{1}H NMR spectra of Li-lipoate before and after curing (30 min) at ambient condition (Fig. S9) ……………………………………………………………………….………. 12

Calculation for the % of polymerization after curing (Li-lipoate) ……………….…..…. 12

^{1}H NMR spectra of Na-lipoate before and after curing (30 min) at ambient condition (Fig. S10) ……………………………………………………………………….…. 13

^{1}H NMR spectra of K-lipoate before and after curing (30 min) at ambient condition (Fig. S11) ……………………………………………………………………….…. 14

A schematic diagram of metal lipoate tissue adhesion (Fig. S12) …………………… 14

A schematic diagram of Zensor electrode set-up in rheometer (Fig. S13) ………….… 14

Table S1. Results of average molecular weight determination by size exclusion chromatography (SEC).

Metal lipoate	Number average molecular weight (M_n)	Weight average molecular weight (M_w)
Li-lipoate	1.02 x 10^5 (27%)	1.02 x 10^5 (27%)
Na-lipoate	5.05 x 10^4 (32%)	1.56 x 10^5 (28%)
K-lipoate	1.41 x 10^5 (27%)	2.03 x 10^5 (28%)

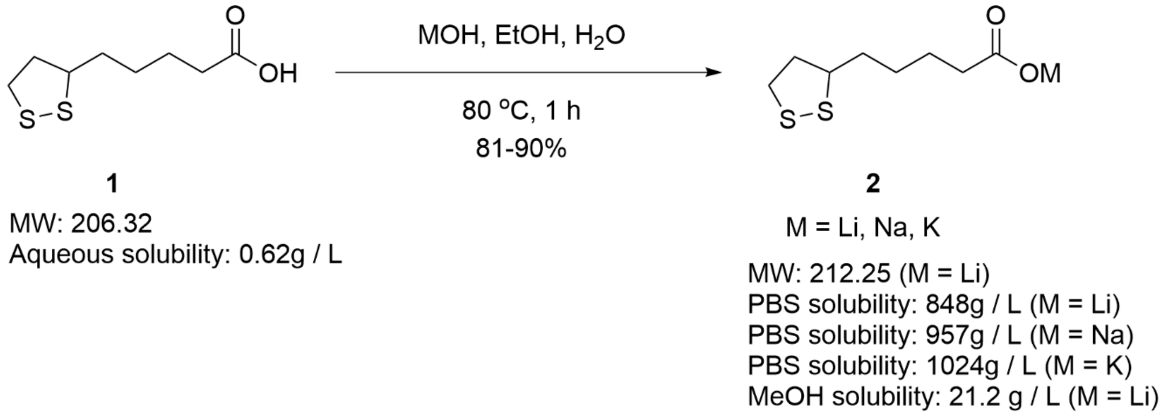

Figure S1. Synthesis of Li-, Na, and K-lipoate. Reagents and conditions: LiOH/NaOH/KOH, EtOH, H_2O, 80 ºC, 1 h, (81–90)%. PBS solubility measured at 24 ºC. Note: after obtaining clear flowable liquid, the solution slowly transitioned to viscoelastic state.

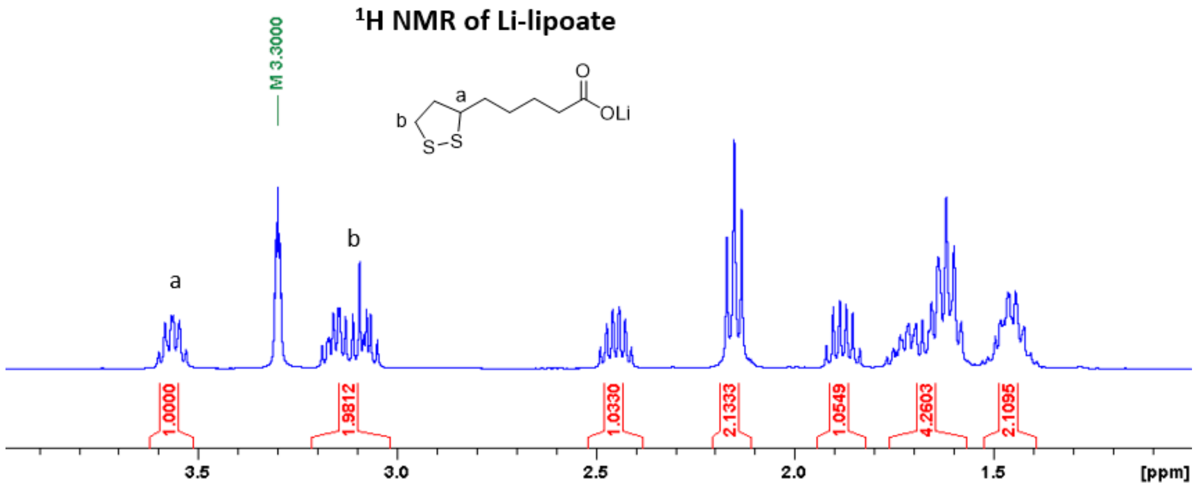
1H NMR of Li-lipoate
M 3.3000
a
b
OLi
O
S-S
1.0000
1.9812
1.0330
2.1333
1.0549
4.2603
2.1095
3.5
3.0
2.5
2.0
1.5
[ppm]

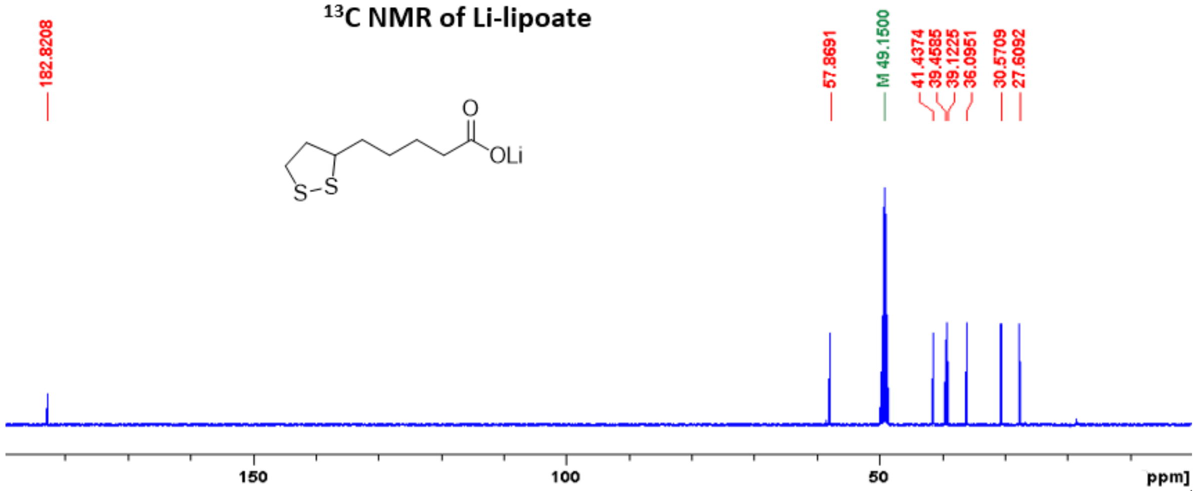
13C NMR of Li-lipoate
182.8208
57.8691
M 49.1500
41.4374
39.4585
39.1225
36.0951
30.5709
27.6092
O
OLi
S-S
150
100
50
ppm]

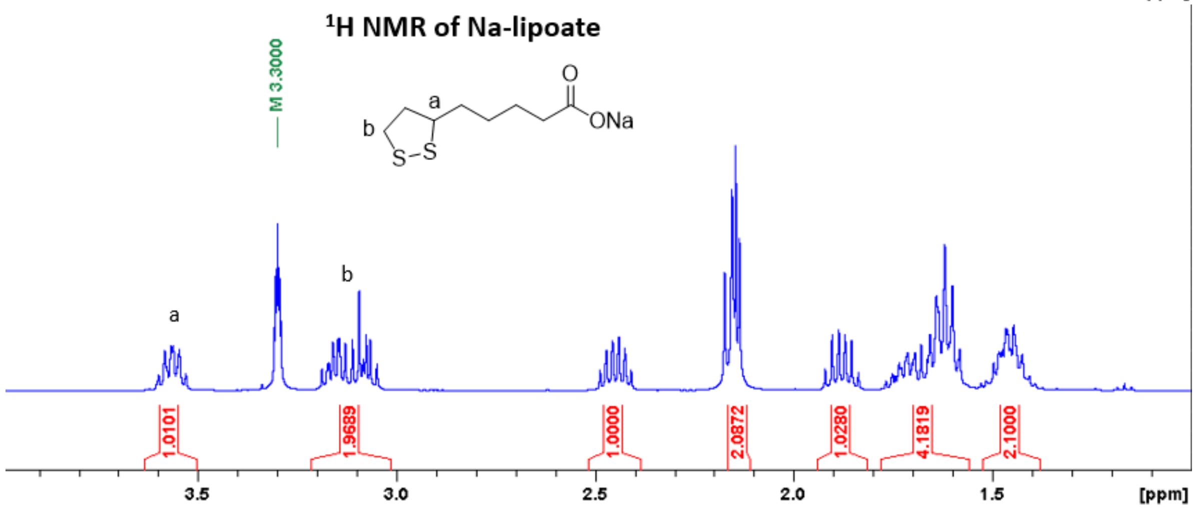
1H NMR of Na-lipoate
M 3.3000
a
b
O
ONa
S-S
1.0101
1.9689
1.0000
2.0872
1.0280
4.1819
2.1000
3.5
3.0
2.5
2.0
1.5
[ppm]

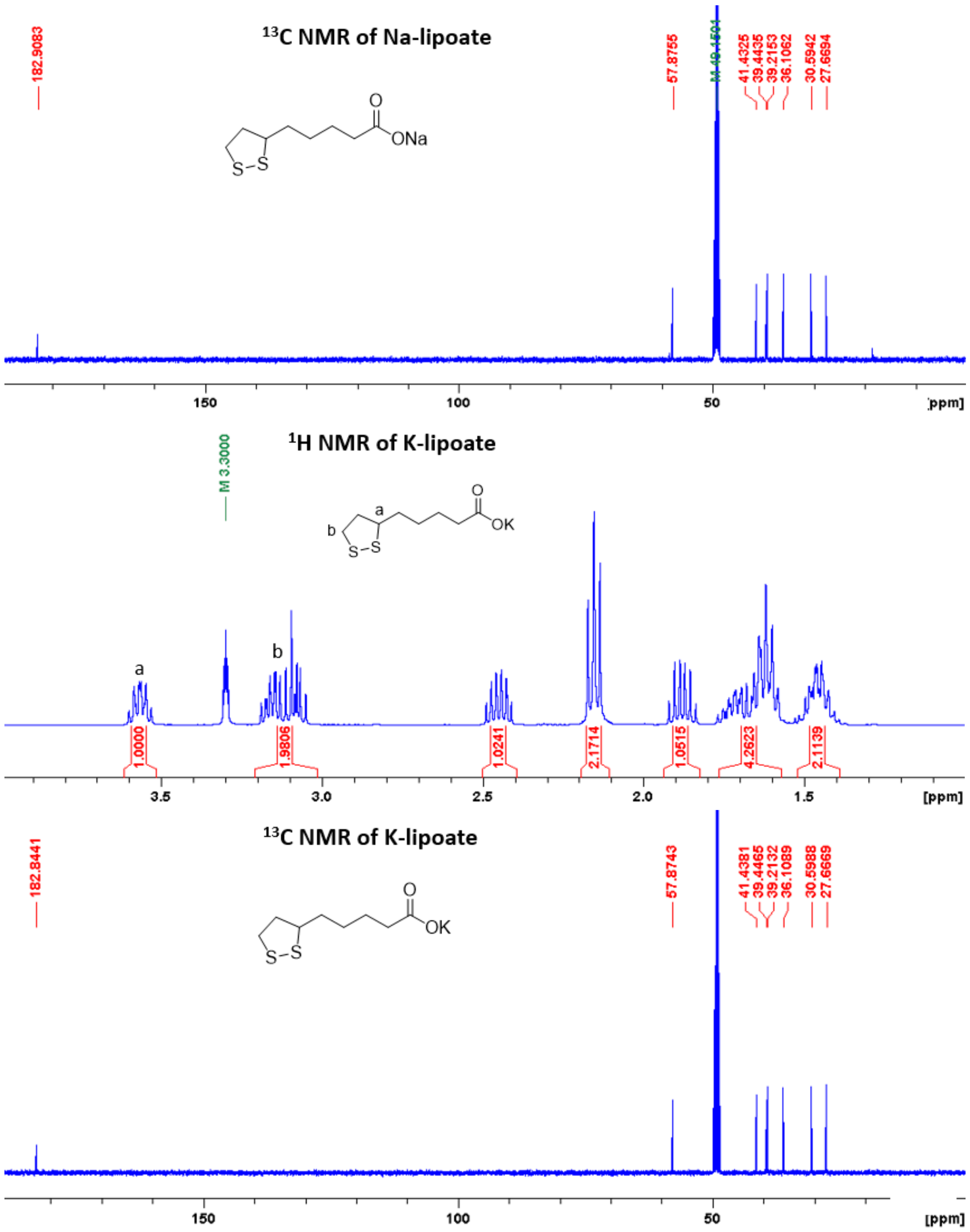

Figure S2. ^{1}H and ^{13}C NMR spectra of (a) Li-lipoate, (b) Na-lipoate, and (c) K-lipoate.

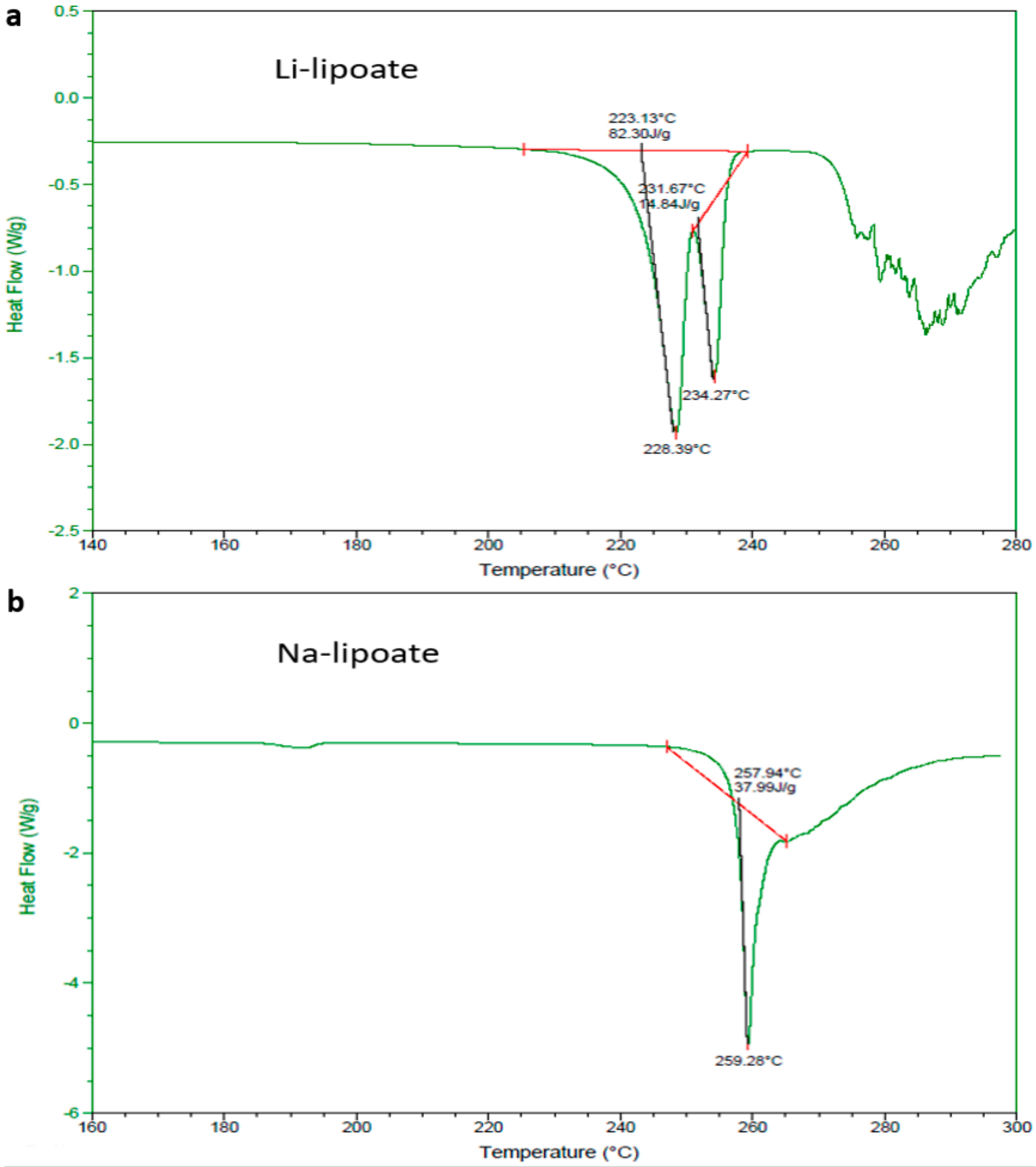
a
Li-lipoate
223.13°C
82.30J/g
231.67°C
14.84J/g
234.27°C
228.39°C
Heat Flow (W/g)
Temperature (°C)
b
Na-lipoate
257.94°C
37.99J/g
259.28°C
Heat Flow (W/g)
Temperature (°C)

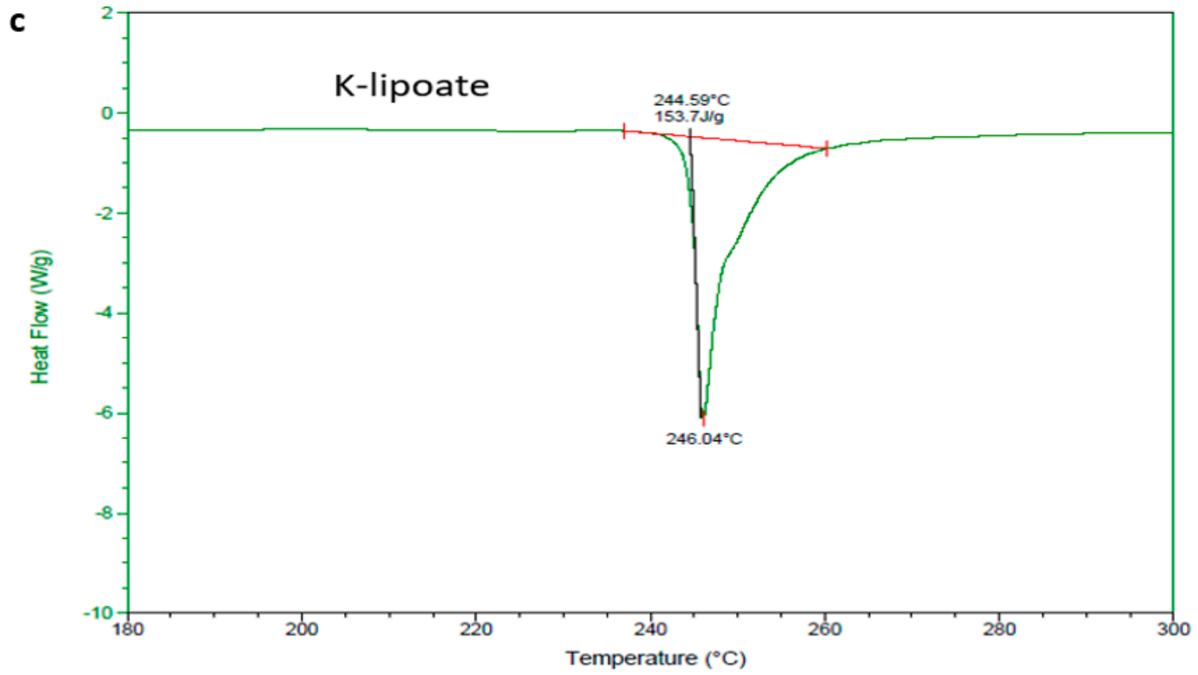

Figure S3. Melting point of (a) Li-lipoate, (b) Na-lipoate, and (c) K-lipoate.

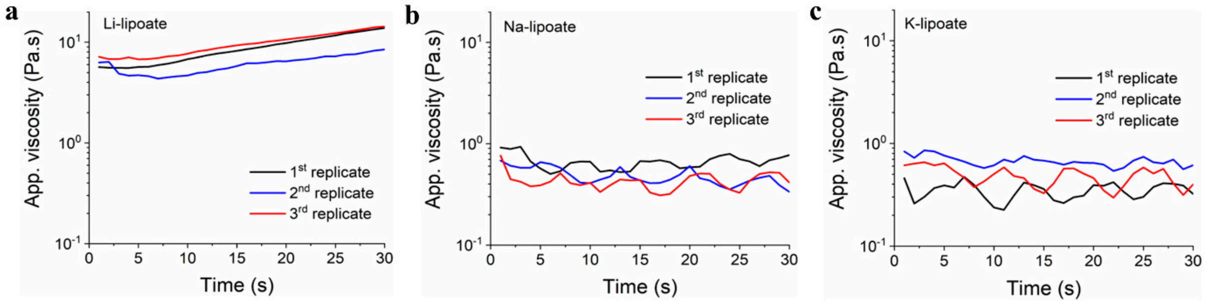

Figure S4. (a) Apparent viscosity of 4M Li-lipoate formulations at fixed shear rate of 10 s^{-1} / 62.8 rad.s^{-1}. (b) Apparent viscosity of 4M Na-lipoate formulations at fixed shear rate of 62.8 rad.s^{-1}. (c) Apparent viscosity of 4M K-lipoate formulations at fixed shear rate of 62.8 rad.s^{-1}.

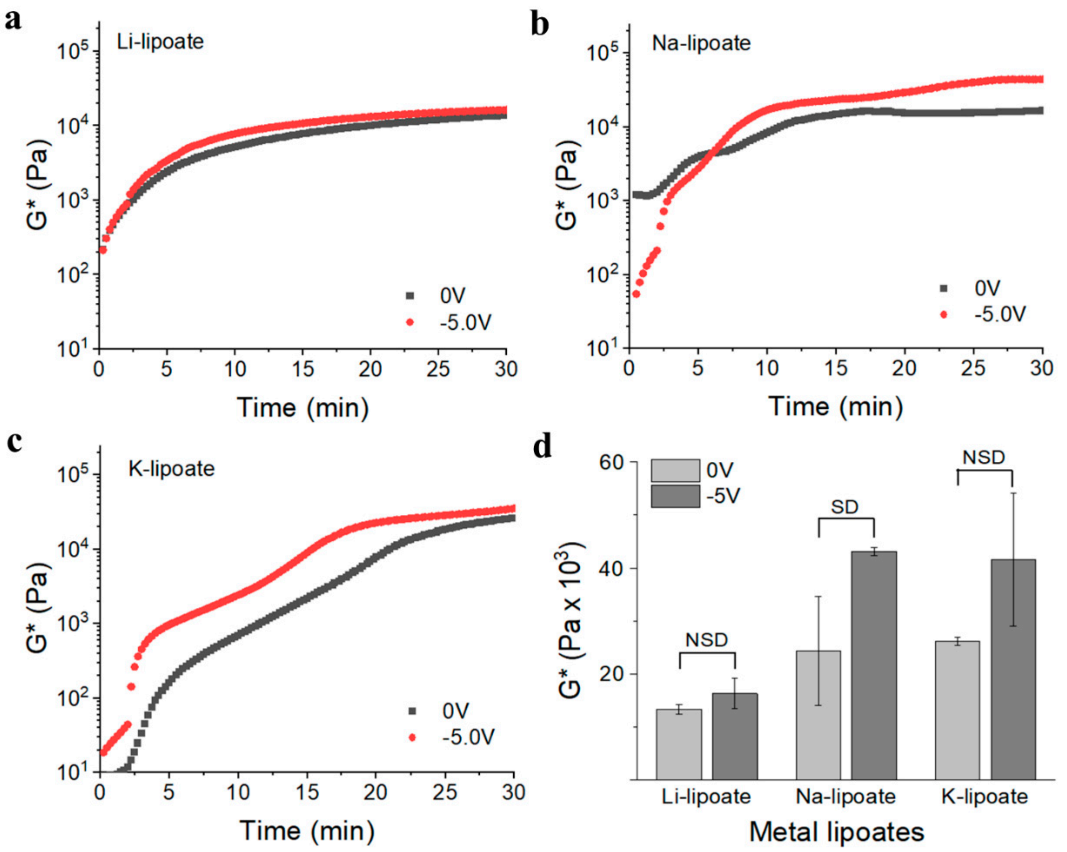

Figure S5. (a) Complex modulus (G*) of Li-lipoate without (0 V) and with voltage (–5.0 V) application. (b) Complex modulus (G*) of Na-lipoate without (0 V) and with voltage (–5.0 V) application. (c) Complex modulus (G*) of K-lipoate without (0V) and with voltage (–5.0 V) application. (d) A comparison of complex modulus (G*) at 0 V and –5.0 V of the three metal lipoates; data presented as mean ± standard deviation, n = 3, *p*-values are calculated using one-way ANOVA with Tukey test, SD (statistical difference) = $p < 0.05$.

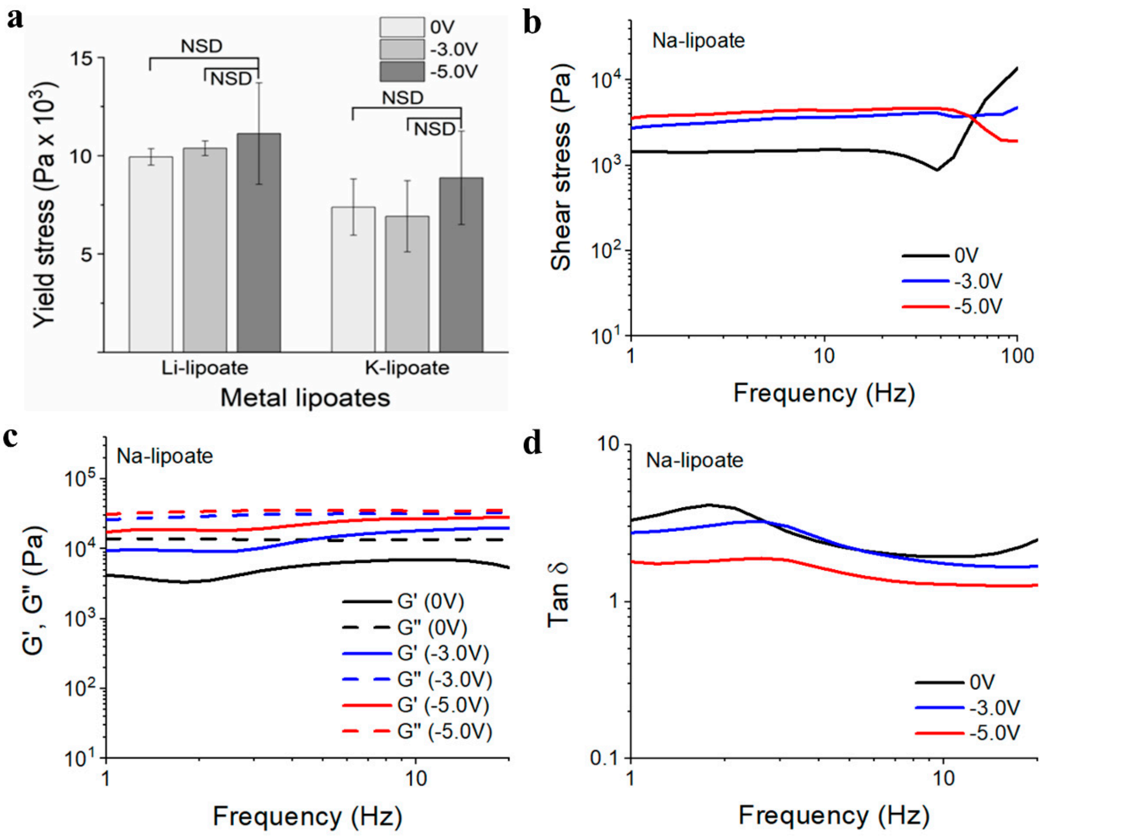

Figure S6. (a) A comparison of yield stress of Li- and K-lipoates at different voltages (0 V, –3.0 V, and –5.0 V). (b) Na-lipoate frequency (1 to 100 Hz) Vs shear stress at different voltages (0 V, –3.0 V, and –5.0 V). (c) Na-lipoate frequency (1 to 100 Hz) Vs modulus (G' and G") at different voltages (0 V, –3.0 V, and –5.0 V). (d) Na-lipoate frequency (1 to 100 Hz) Vs Tan δ (G' and G") at different voltages (0 V, –3.0 V, and –5.0 V).

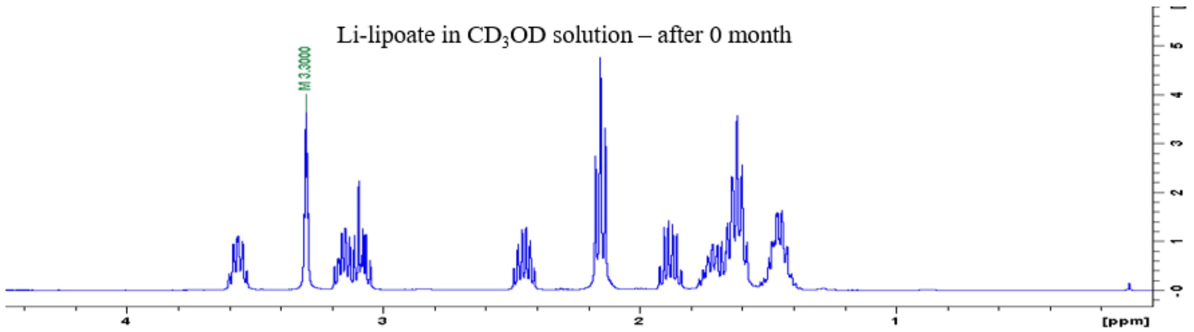
Li-lipoate in CD_3OD solution – after 0 month
M 3.3000
4
3
2
1
[ppm]

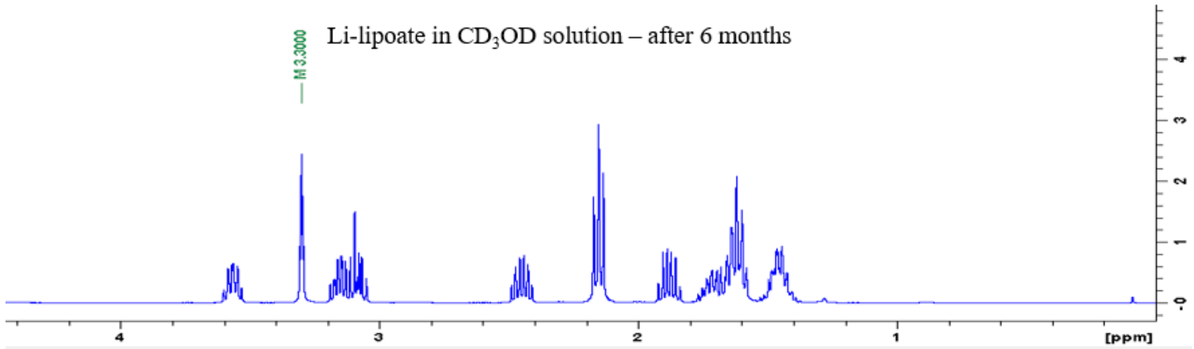
Li-lipoate in CD_3OD solution – after 6 months
M 3.3000
4
3
2
1
[ppm]

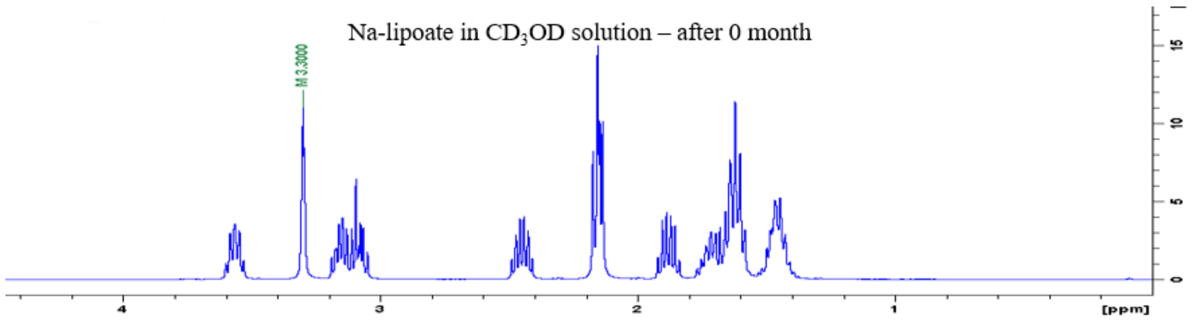
Na-lipoate in CD_3OD solution – after 0 month
M 3.3000
4
3
2
1
[ppm]

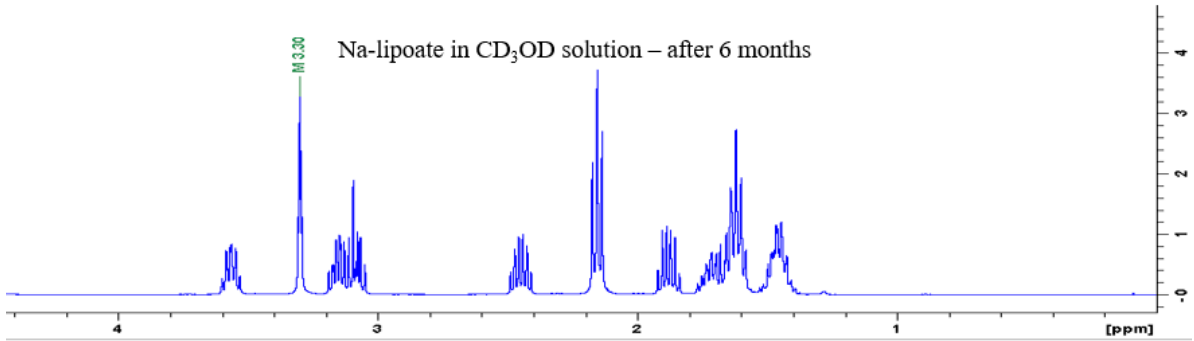
Na-lipoate in CD_3OD solution – after 6 months
M 3.30
4
3
2
1
[ppm]

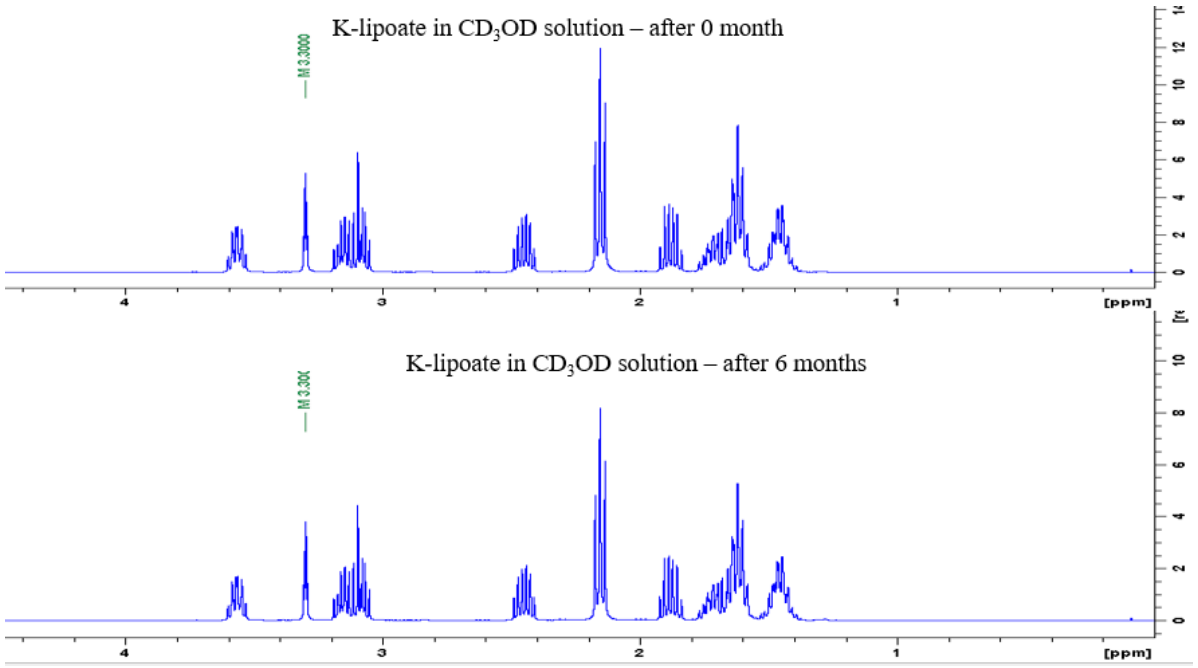

Figure S7. ^{1}H and of (a) Li-lipoate, (b) Na-lipoate, and (c) K-lipoate in CD_3OD solution stored at room temperature up to 6 months.

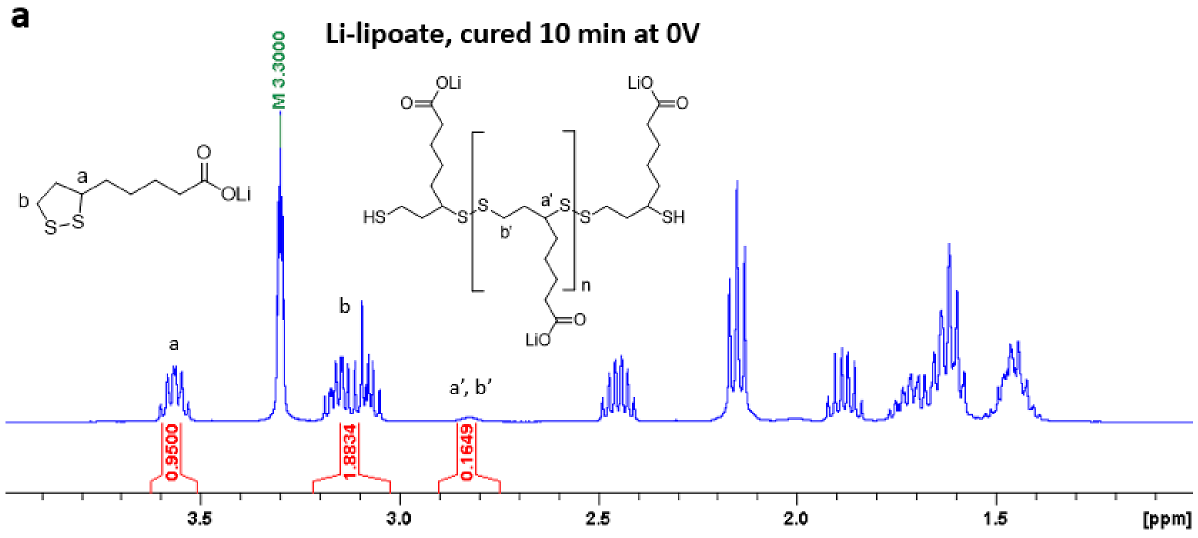

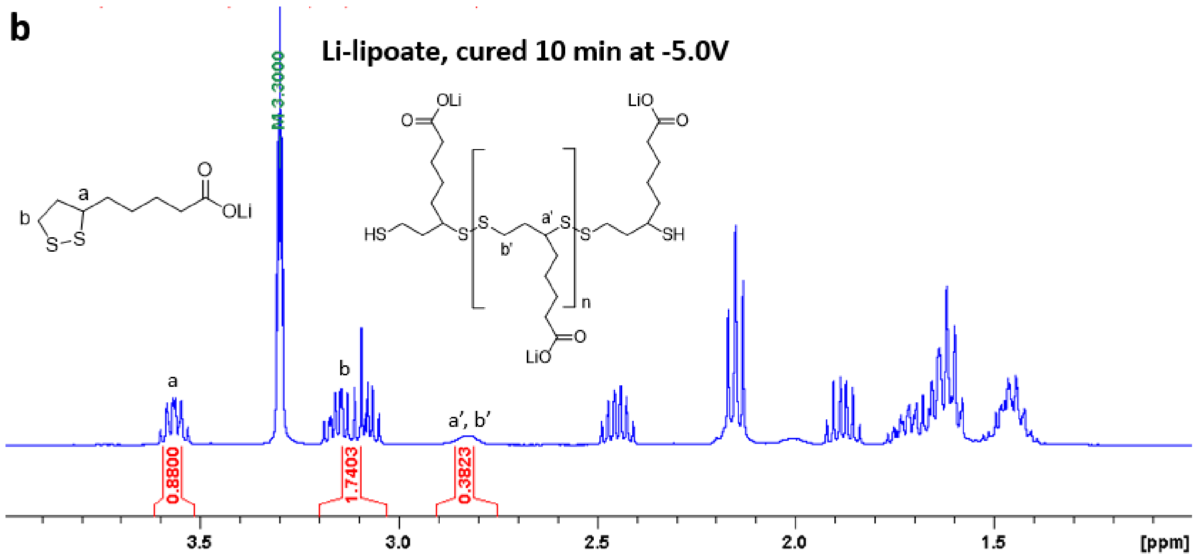

Figure S8. Comparison of ^{1}H NMR spectra of Li-lipoate at (a) 0 V shows 5.5 % monomer conversion and (b) at –5.0 V shows 12.7 % monomer conversion after 10 minutes curing.

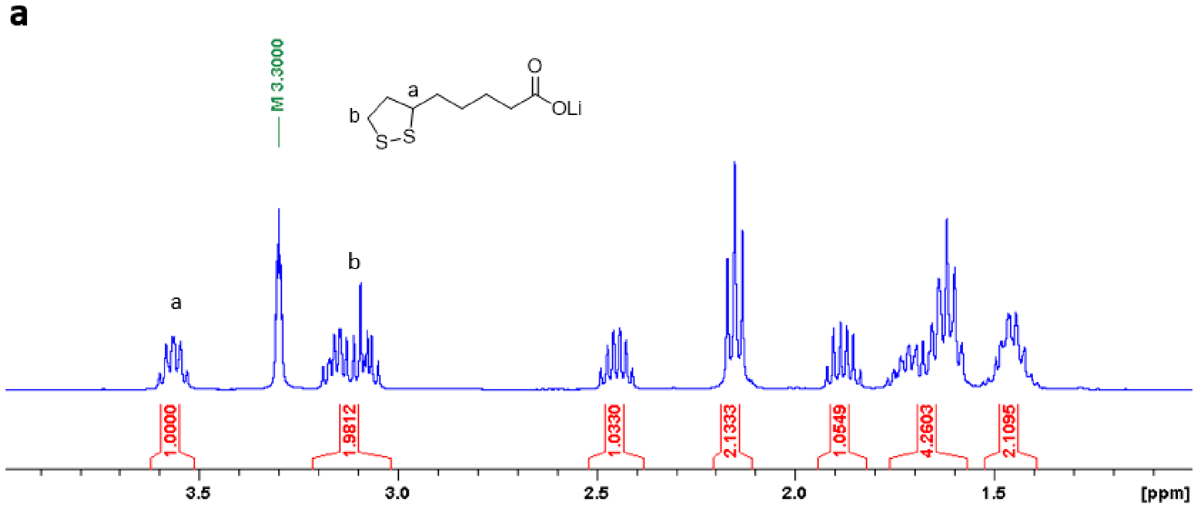

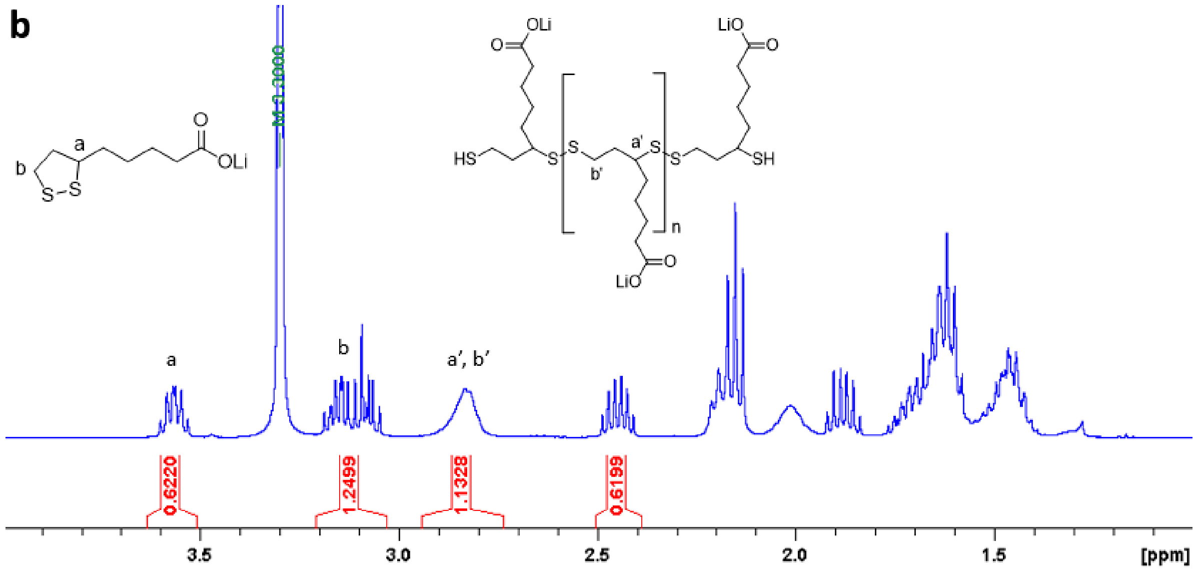

Figure S9. ^{1}H NMR spectra of Li-lipoate (a) before curing and (b) after 30 min curing at ambient condition. % of Li-lipoate polymerization = 37.6%.

Calculation for the % of polymerization after curing (Li-lipoate):

Before curing there was no peak at 2.83 ppm. New peak (a' and b') originates from the CH and CH_2 protons (a and b) directly attached to sulfur atoms in the 5 membered ring after formation of the linear polymer.

Integration of peaks a and b before polymerization = 3.0

Integration of new peaks a' and b' = 1.13

% of Li-lipoate polymerization $= \frac{1.13}{3.0}$ x 100 = 37.6%

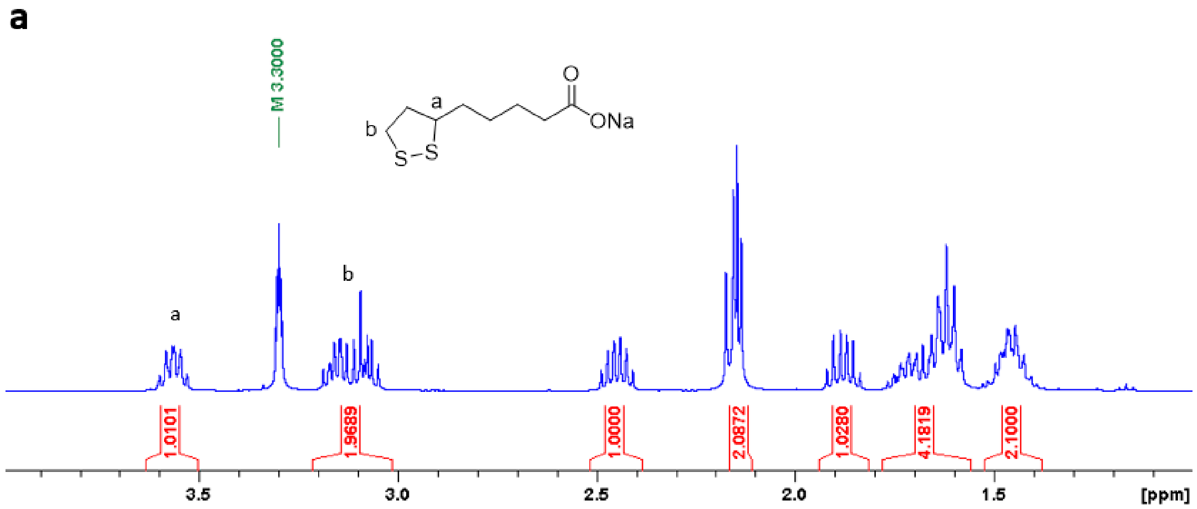

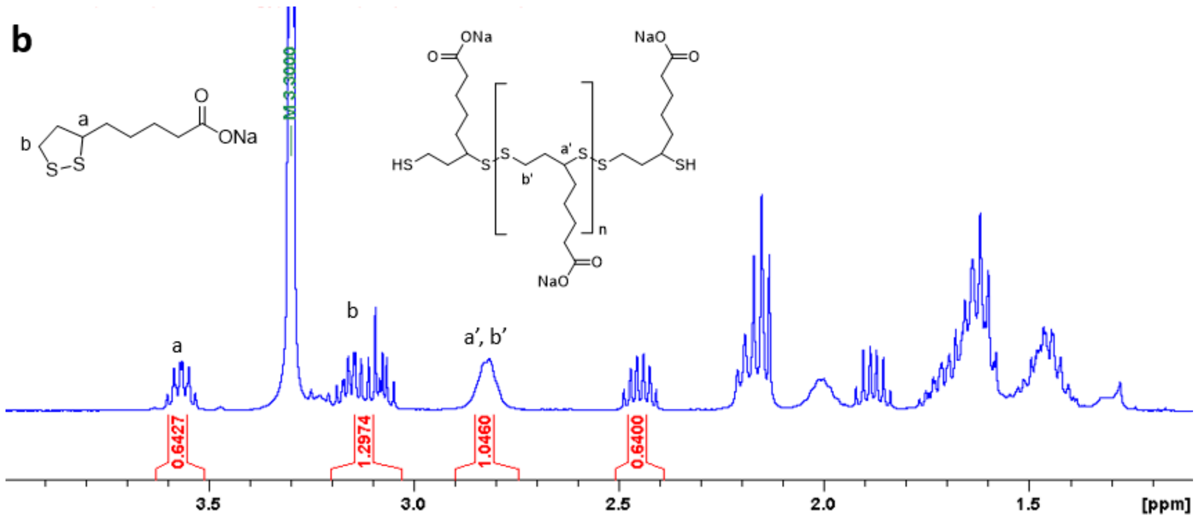

Figure S10. ^{1}H NMR spectra of Na-lipoate (a) before curing and (b) after 30 min curing at ambient condition. % of Na-lipoate = 34.7%

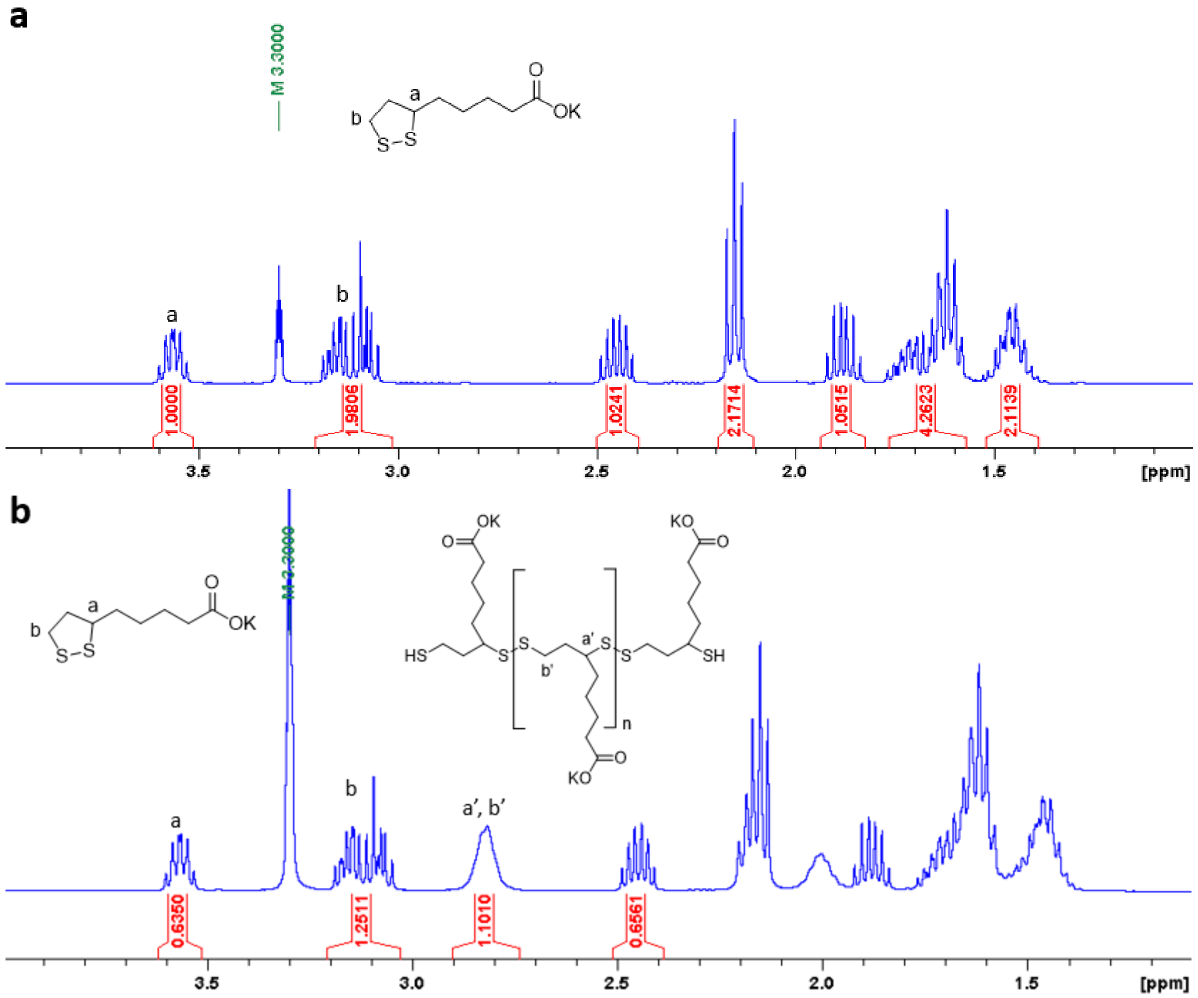

Figure S11. ^{1}H NMR spectra of K-lipoate (a) before curing and (b) after 30 min curing at ambient condition. % of K-lipoate polymerization = 36.6%.

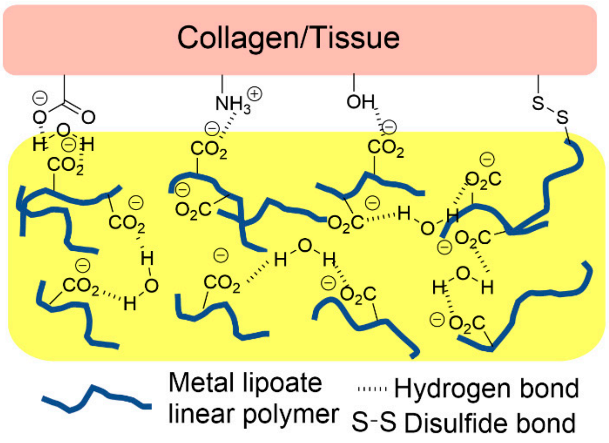

Figure S12. A schematic diagram of metal lipoate tissue adhesion.

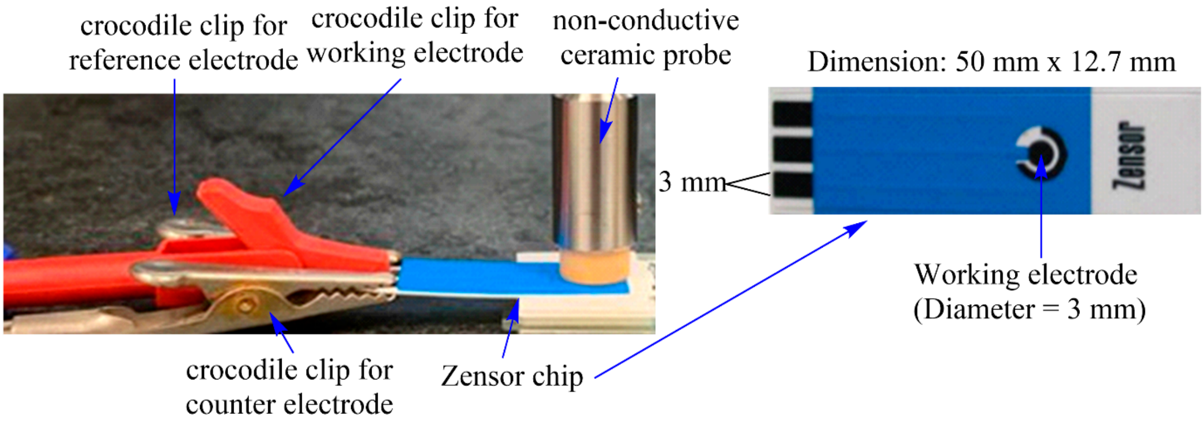

Figure S13. A schematic diagram of Zensor electrode set-up in rheometer.
